# Randomized, Placebo-Controlled Prospective Clinical Trial Evaluating the Efficacy of the Assisi Anti-anxiety Device (Calmer Canine) for the Treatment of Canine Separation Anxiety

**DOI:** 10.3389/fvets.2021.775092

**Published:** 2021-12-20

**Authors:** Katherine Pankratz, Judy Korman, Carrie Emke, Brianna Johnson, Emily H. Griffith, Margaret E. Gruen

**Affiliations:** ^1^Behavioral Medicine Service, Department of Clinical Sciences, College of Veterinary Medicine, North Carolina State University, Raleigh, NC, United States; ^2^Assisi Animal Health, Northvale, NJ, United States; ^3^Clinical Studies Core, Department of Clinical Sciences, College of Veterinary Medicine, North Carolina State University, Raleigh, NC, United States; ^4^Department of Statistics, College of Sciences, North Carolina State University, Raleigh, NC, United States; ^5^Comparative Behavioral Research, Department of Clinical Sciences, College of Veterinary Medicine, North Carolina State University, Raleigh, NC, United States

**Keywords:** dog, pulsed electro-magnetic field (PEMF), behavior, fear, separation distress, separation-related behaviors

## Abstract

**Introduction:** Separation anxiety (SA) is among the most common canine behavior disorders and affects quality-of-life for dogs and their owners. Dogs with SA show signs of anxiety during absence or perceived absence of their owners. While psychoactive medications are often helpful for treating SA, dog and human factors may limit their utility. This study explored the efficacy of a pulsed electromagnetic field (PEMF) device for treatment of canine SA.

**Materials and Methods:** In this double-blind randomized placebo-controlled study, a screening questionnaire and baseline video confirmed the diagnosis of SA. Owners treated their dog with the device twice daily for 6 weeks, completed weekly questionnaires, and noted adverse events. Videos were taken of the dog while alone at weeks 4 and 6. Behaviors were coded and categorized as negative and positive. Questionnaire and video data at weeks 4 and 6 were compared to baseline.

**Results:** Forty client-owned dogs with moderate to severe SA completed the study. There were no differences between groups for age, weight, or sex. In owner questionnaires, no difference in behavior or overall score was found between the active and sham groups (*p* > 0.05). Videos of the active group compared to the sham group showed significant reduction in negative behaviors by week 6 (*p* = 0.036) and higher percentage of success at week 4 (*Z* = 2.83, *p* = 0.005), at week 6 (*Z* = 1.65, *p* = 0.098), and across the full study (*Z* = 1.99, *p* = 0.047). Adverse events were reported in eight dogs (6 active, 2 sham); all resolved and were unlikely to be related to treatment.

**Discussion:** This study supports the efficacy and safety of this PEMF device for treatment of SA in dogs. Questionnaire results may not be sensitive enough to detect subtle negative behavioral states compared to video, and may not capture other owner observed behavioral changes. A caregiver placebo effect may account for some improvement seen in both groups. Video data appear better for diagnosis and monitoring dog's behavior when left alone. Future studies should assess PEMF's impact on other anxieties or combination of anxiety disorders in dogs.

## Introduction

Separation anxiety (SA) in dogs is a common behavioral disorder, affecting between 14 and 20% of the pet dog population (1, 2). Several terms, including separation-related behavior and separation-related distress (3–5) have been used to describe this condition, which is characterized by signs of fear and anxiety and during real or perceived separation from an attachment figure. Commonly described signs of SA include destructiveness, vocalization, hypersalivation, and housesoiling during separations; these more “active” signs can be easier for owners to detect. However, video analyses of dogs with SA have shown that these dogs also spend a significant proportion of their time in stationary orientation to their environment without interaction with toys or food (6, 7). This is compared to dogs without SA, who spend the majority of their time in passive rest (8). As SA requires the owner to be away from the dog, the high proportion of time spent orienting to the environment can only be appreciated through video, making this essential for both the diagnosis and monitoring of treatment success.

Correct diagnosis is critical as SA often represents a welfare concern for dogs. It also represents a significant burden for their owners, with recent work highlighting the negative emotions and strain on relationships experienced by owners of dogs with SA (9). Treatment plans for SA involve behavioral and environmental modification and often include psychoactive medication for managing anxiety. Two medications have been approved in the United States for the treatment of canine SA: fluoxetine (Reconcile, PRN Pharmacal) and clomipramine (ClomiCalm, Novartis AG). Both of these medications showed efficacy in clinical trials (10–12) with improvement in signs of SA over placebo. However, across these trials (and depending on the timepoint) up to 35% of dogs showed no improvement while on treatment (10, 11). In addition, some dogs may not tolerate the medications, may not have their SA sufficiently controlled, or owners, for a variety of reasons, may wish for a non-pharmacologic treatment option (13).

Pulsed electro-magnetic field (PEMF) therapy has the potential to offer a non-pharmaceutical option or addition to therapy for SA. Targeted PEMF has a long history of use for treatment of non-union fractures, wound healing, edema, post-operative and osteoarthritic pain in humans (14) and animals (15–17). Pulse generators create alternating magnetic fields that modulate neuronal excitability but are below the threshold to induce action potentials. PEMF is believed to increase calcium signaling and have anti-inflammatory effects through the generation of nitric oxide (18, 19). Recently, more attention has been turned to the potential use of PEMF in psychiatric conditions. Work in rodents has shown efficacy for PEMF in reducing anxiety behaviors and increasing neurogenesis in the hippocampus in a model of post-traumatic stress (20), however other work has shown no change in anxiety responses following exposure to electromagnetic fields (21). In humans, PEMF has shown rapid positive effects on mood in depressed individuals (22, 23) and longer-term positive effects on those with treatment resistant depression (24).

Until recently, no work had been done evaluating PEMF therapy for anxiety in veterinary clinical settings. In 2019, a pilot study was conducted with 10 canine patients with SA (25). Owners administered PEMF treatments twice daily for 6 weeks, completed questionnaires every 2 weeks, and video-recorded their dogs home alone on weeks 4 and 6. Results of owner questionnaires were positive, with all enrolled patients improved by 4 weeks, and signs in more than half of patients resolved by 6 weeks. At both 4 and 6 weeks, videos of the dogs when home alone showed an increase in positive behaviors such as resting, and a decrease in negative behaviors such as pacing and orienting to the environment when compared to baseline videos. However, this study lacked a control group, making a more rigorously designed study necessary to draw conclusions on efficacy. Thus, the objectives of the current study were to evaluate the safety and efficacy of a PEMF device in a randomized, sham-device-controlled clinical study. If helpful for the treatment of SA, we would predict that dogs in the active device group—relative to the sham device group—would have lower anxiety scores on owner-reported questionnaires and would show increased positive behaviors and decreased negative behaviors on video when left home alone.

## Methods

### Study Devices and Blinding

The pulsed electro-magnetic field (PEMF) device was portable, handheld, and battery operated with a pulse rate of 7 bursts per second, at a frequency of 27.12 MHz and a peak induced magnetic field of 4uT.^1^ Treatments were administered by positioning the pulse generator near the base of the skull (occipital bone), pushing the button, and holding it in place with the coil positioned over the skull for the duration of the 15-min treatment; a blinking green light signaled that the device was working. Sham-devices were identical, including with the blinking green light, however did not generate electromagnetic pulses (Supplementary Figure 1). Devices were labeled with a device number, and were distributed to owners based on enrollment in a randomized order. Study staff, investigators, and the study statistician were blind to group designation until analyses were complete.

### Study Population

Recruitment began in February, 2019. Dogs were initially recruited from the area surrounding the North Carolina State University College of Veterinary Medicine; in August, 2019, recruitment was expanded to a national sample to increase enrollment. All procedures were approved by the NCSU Institutional Care and Use Committee (Protocol #18-174-O) and all owners provided written informed consent for the study.

Dogs were eligible for the study if they were naïve to the PEMF device, between 1 and 13 years of age, generally healthy (based on physical exam and routine medical care), had clinical signs consistent with SA for at least 3 months prior to enrollment, and were classified as moderate or severe (based on outcome measures as described below). On a 60-min screening/baseline video of the dog left alone, dogs had to be visible for a minimum of 15 min of the first 30 min of the video, and must be engaged in negative behaviors associated with SA (described below) for a minimum of 5 min. Dogs were still eligible if they were taking one psychoactive or sedating medication, provided that the dose had been stable for at least 1 month, signs of SA were still severe enough to meet entry criteria; dosage changes were not allowed once enrolled in the study. Dogs were excluded if they were pregnant or lactating, had a diagnosis of thunderstorm or noise phobia or suspect confinement distress in addition to SA, if they were receiving more than one psychoactive or sedating medication, or if they were receiving a psychoactive or sedating medication whose dose had not been stable for at least 1 month.

Owners were eligible for the study if they were able to provide a stable home environment for the duration of the study [no major changes in household routine, vacation longer than 3 days without the dog, extended guest stays, or change in family members in the home (including new pets)]. Owners also had to commit to being compliant with the treatment administration, and had to leave their dog alone for at least 60 min, at least three times per week. This requirement was to ensure that owners would have sufficient opportunities to evaluate their dog. Owners also had to agree not to start any new psychoactive medications, supplements, or other modalities for the treatment of SA during the study.

Dogs were screened via a medical and behavioral history, and review of medical records. A physical exam was performed for enrollment, either by the study investigators or, in the case of non-local participants, by the patient's primary veterinarian. Owners of eligible dogs were sent a video camera and SD card to record a 60-min departure, leaving their dog in the location and setting that they normally would; owners could also record remotely over a cloud-based system. Once recorded, the SD card was returned and the video was reviewed by a board-certified veterinary behaviorist to ensure inclusion criteria were met. If so, owners were sent additional SD cards and a device (active or sham) as well as a diary to note treatment and instructions for the study.

Owners administered the treatment for 15 min twice daily—at least 8 h between each treatment—and recorded treatments on their study diary. Owners held the study device centered over the dog's occipital bone (either resting on the head or held one to three inches from the head) during treatment administration; dogs were not confined during treatment. Owners completed outcome assessments (questionnaires and video) as described below. At the completion of the study, the devices were returned to NCSU-CVM. After the first 40 dogs completed the study, the devices were sent to the manufacturer for testing; devices that were no longer active were removed from the study and replaced. Throughout the study, owners monitored for any adverse events, and were instructed to report them to the investigators if noted.

### Outcome Measures

#### Questionnaires

At baseline, and weekly after enrollment, owners were sent online questionnaires through Qualtrics (Qualtrics, Provo, UT). On these questionnaires, owners were asked to rate their dog's behavior on a 4-point continuous scale from 0 = absent to 3 = severe for each of 5 behaviors (destructive behavior, rearranging, excessive vocalization, inappropriate urination, and inappropriate defecation). In order to be eligible for inclusion, dogs had to have a score of 2 or higher in at least two behaviors. Owners also assigned an overall (global) score for their dog's SA using the same 4-point continuous scale. In order to assess behaviors specific to their dog, owners were also asked to identify 3 behaviors shown by their dog, and rated the severity of each of those behaviors using the same 4-point continuous scale. After enrollment, owners were also asked about any concerning signs or adverse events, and given space to provide any feedback they had about the study.

#### Video

In addition to the baseline video, owners were instructed to record a 60-min video of their dog during a routine departure on Days 28 (week 4) and 42 (week 6). As with the baseline video, these could be recorded on an SD card or via the cloud-based system; SD cards were then sent to the study investigators.

To assess the dog's behavior when left alone, behaviors were coded from video using an ethogram adapted from Cannas et al. (7). Durations or frequencies of behaviors were calculated using the descriptions shown in Table 1. Behaviors were classified as positive or negative (associated with SA) as described, and based on descriptions used in other published studies (6–8). Passive behavior (generally sleeping or resting) and interacting with the environment (normal interactions with toys, food, etc.) were used as indicators of positive behavior; passive behavior has been shown to be the predominant behavioral state of adult dogs without SA when home alone (8) and an increase in passive behavior has been used as an indicator of treatment efficacy (7). Negative behaviors included those typically associated with separation anxiety (destruction, rearranging, vocalizing, restlessness/pacing, and orienting to the environment) (6–8).

**Table 1:** Description of the behaviors observed in the video recordings.

**Duration Behaviors (time)**	**Definition**
**Positive Behaviors**
Interaction with the environment (IE)	Locomotive or stationary interaction directed at exploring the environment (i.e., chewing a bone, drinking water, eating food)
Passive behavior (PA)	Lying down with the head on the ground without any obvious orientation toward the physical or social environment without vocalizing
**Negative behaviors**
Destructive/Active (DE)	Destructive, active behaviors directed at the environment including scratching, jumping, biting objects such as doors, windows, or gates
Rearranging behavior (RA)	Manipulating objects in the environment, moving with nose or paws, but without destruction
Restlessness/Pacing (RP)	Active movement such as restlessness, pacing, circling, and other locomotion not directed at exploring the environment
Oriented to the environment (OE)	Stationary such as sitting, standing, or lying down where the head does not rest on the ground. There is obvious orientation with the eyes open toward the physical or social environment such as close visual inspection, distant visual inspection (vigilance or scanning)
Whining/Howling (WH)	Vocalizations that include whining, howling, or continuous vocalizations
Not visible (NV)	Not visible. If audible sounds like barking, whining, scratching, or chewing were identified, they are recorded by the sound of the activity
**Frequency Behaviors (counts)**	**Definition**
Barking (BA)	Barking (individual vocalization of a sharp explosive cry)
Yawning (YA)	Yawning (opening the mouth wide while inhaling deeply)
Elimination of urine (EL Urine)	Urination in sitting or standing position
Elimination of feces (EL Feces)	Defecation in sitting or standing position

*Duration behaviors were coded as time (minutes and seconds), and categorized as positive behaviors when the dogs were resting quietly or interacting normally with the environment, and negative behaviors when dogs were showing signs associated with anxiety. Frequency behaviors were coded as counts*.

Videos were coded by four independent observers, all blind to the treatment group, with 24 videos (20%) coded by multiple raters to evaluate interrater reliability. For consistency within a case, all three videos for a given dog were coded by the same observer.

### Statistical Analysis

The statistical analyses of the questionnaire and video data were performed using SAS software (version 9.4, Cary, NC). The analyses were run on blinded data, with treatment groups marked as A and B. The data were unblinded after the results were run. A few subgroup analyses were run after the unblinding and are specifically noted.

The baseline measurement was subtracted from each week of questionnaire measurements. A new variable was created for a two-point improvement for each week of the questionnaire (defined as a success). At each week, Fisher's exact tests were run on contingency tables comparing the active to sham device and two-point improvement. Additionally, a logistic regression analysis was run that included device (active or sham), sex (m/f), and medication (y/n). These models were run for each individual behavior, the overall score, and the sum of the scores of the behaviors specified by owners.

Inter-rater reliability was evaluated by calculating intraclass coefficients for each coded behavioral state and event. The video data were analyzed using a repeated-measures ANCOVA in order to explore both the time trends within dogs and the device effects between dogs (26). The model included the baseline value as a covariate, a random subject (dog) effect, a device effect, a time effect [week 4 (day 28), week 6 (day 42)], and a device by time interaction term. The response variable was the difference between the week 4 and 6 value and the baseline value to allow us to directly estimate the change in behavior due to the device while still adjusting for baseline values to avoid regression to the mean (27). The covariance matrix was compound symmetric and was chosen using Akaike's Information Criterion. When residual diagnostics indicated that there was heterogeneity in the variance, natural-log transformations of the response variable were performed and models were re-fit. Least-squares means and confidence intervals were calculated for each device by date combination. An additional analysis was performed on a dichotomized response using 100% increase in positive behavior as the minimum criteria for success. The difference in the proportion of successes in each treatment group was compared using a two-sided z-test (28).

In order to compare the video and questionnaire data, Pearson's correlations were calculated for corresponding behaviors across the full study and by week.

After the study was unblinded, a repeated-measures model was fit for only the dogs in the active group to examine the impact of sex, weight, and age on the percentage of time engaged in positive behavior as well as the percentage of time engaged in negative behavior.

## Results

### Participants

Dogs ranged in age from 1.1 to 11.3 years of age and in weight from 2 kg to 38.3 kg. Demographics are shown in Table 2; no differences were found between groups with regard to age, weight, or sex. Six dogs in the active group were receiving one psychoactive medication [fluoxetine (2), clomipramine (2), trazodone (1), alprazolam (1)]; three dogs in the sham group were receiving one psychoactive medication [fluoxetine (2), trazodone (1)]. No dogs were receiving more than one psychoactive medication.

**Table 2:** Demographics of participating dogs.

**Variable**	**Sham**	**Active**	**Test statistic and *p*-value**
Age (mean +/- SD)	5.8 (3.1)	4.8 (2.1)	*t* = 1.22; *p* = 0.230
Weight (mean +/-SD)	17.1 (10.4)	17.2 (8.8)	*t* = 0.0475 *p* = 0.962
Sex	7 female (1 FI, 6 FS), 13 male (2 MI, 11 MC)	9 female (all FS), 11 male (all MC)	Fisher's exact test; *p* = 0.747

*There were 20 dogs in the sham group and 20 dogs in the active group*.

### Outcome Measures

#### Weekly Questionnaires

To evaluate improvement in each treatment group, overall scores and scores for each behavior were evaluated. Success was defined as a 2-point improvement in owner score from baseline. While both groups were significantly improved from baseline, no difference was found between treatment groups for the distribution of successes for any individual behavior (destructive, rearranging, vocalization, urination, defecation), the overall score at week 4 or week 6, or for the behaviors specified by owners (all *p* > 0.05; Supplementary Table 1). Finally, logistic regression models failed to converge for most variables, with the exception of “defecation” where there was no effect of group, patient sex, or whether they were on psychoactive medication on the distribution of success.

#### Video Data

Across all dogs and timepoints, mean video length was 53.3 min (+/-13.4 min) with a mean of 2.9 (+/- 4.8) min where the dog was not visible. However, at week 4, the minimum video length was 3.0 min, and at week 6 the minimum video length was 13.6 min. As SD cards were mailed back to the investigators, the delay between the video acquisition and review meant that these could not be replaced for those timepoints. There was a strong negative correlation between the video length and the time not-visible (-0.98,−0.97,−0.99 for baseline, week 4, and 6, respectively). Intraclass coefficients for each behavioral state and event were generally good (0.81–0.85) or excellent (0.90–0.97), with the exception of “interacting with the environment” which was poor (0.45), and “yawn” and “restless/pacing” which were moderate (0.56 and 0.67, respectively) (29). Intraclass coefficient results are shown in Supplementary Table 2; importantly, the correlation between “passive” and “orienting to the environment” was excellent (0.90 and 0.92, respectively).

#### Change in Negative Behaviors

As not all dogs displayed all negative behaviors, a sum score was created for each dog that included the percentage of time spent in the behavioral states of: destruction, rearranging, restless/pacing, and orienting to the environment (referred to as “negative behaviors”). There was no significant effect of time or treatment at the 0.05 level; there was a treatment effect at the 0.10 level. The difference in mean log-response results between the two treatment groups was not significantly different at week 4 (difference = 6.67 +/- 6.84, *t* = 0.98, *p* = 0.336), but was significantly different at week 6 (difference = 16.70 +/- 7.65, *t* = 2.18, *p* = 0.036; Table 3).

**Table 3:** Results of repeated-measures ANOVA evaluating treatment (sham vs. active), and time effects.

**Effect**	**F-test degrees of freedom**	**F Value**		**Pr > F**
Treatment	1, 35.3	3.77		0.060
Day	1, 35.5	0.00		0.953
Treatment*Day	1, 35.5	1.53		0.224
Baseline	1, 35.8	3.37		0.074
**Label**	**Estimate**	**Degrees of freedom**	***t*** **-Value**	**Pr** **>** **|t|**
Week 4: Sham vs Active	6.668	35.4	0.98	0.336
Week 6: Sham vs Active	16.695	35.3	2.18	0.036

*The difference in expected mean log-response is shown between the two treatment groups at weeks 4 and 6. These differences are calculated as sham minus active. The difference at week 6 is statistically significant, with sham being higher (more negative) than active*.

Expected mean difference with 95% confidence intervals are shown for the comparison between the week and baseline value of the response at each time and treatment combination (Table 4). The mean difference is significantly different from zero for both sham and active treatments at week 4, and just for the active treatment at week 6.

**Table 4:** Expected mean difference and 95% confidence intervals for the comparison between week (4 or 6) and baseline for all negative behaviors recorded on the videos.

**Least Squares Means**
**Effect**	**Treatment**	**Week**	**Estimate**	**Standard error**	**Lower 95% Confidence interval**	**Upper 95% Confidence interval**
Treatment*Week	Sham	4	−10.955	4.823	−20.742	−1.167
Treatment*Week	Sham	6	−5.702	5.315	−16.496	5.092
Treatment*Week	Active	4	−17.622	4.812	−27.389	−7.856
Treatment*Week	Active	6	−22.397	5.451	−33.461	−11.333

### Success/Failure

Success/failure was evaluated using change in positive behaviors: a dog was categorized as a success if the sum of their positive behaviors (IE + PA) increased by 100% or more from baseline. Distributions for successes at each week, separated by treatment, are shown in Table 5. At week 4, 20% of dogs in sham group and 60% of dogs in active group were categorized as successes, with a higher percentage of success in active group (*Z* = 2.83, *p* = 0.005). At week 6, 30% of dogs in sham group and 55% of dogs in active group were categorized as successes; the proportions were different at the 0.10-level (*Z* = 1.65, *p* = 0.098). Across the full study, 35% of dogs in the sham group and 65% of dogs in active group were categorized as successes, with a significantly higher percentage of successes in the active group (*Z* = 1.99, *p* = 0.047).

**Table 5:** Distributions for the number of dogs in categories of success/failure (defined as an increase of 100% or more in positive behaviors compared to baseline) separated by treatment (sham or active) and time (week 4, 6, and overall across the study).

	**Success (Week 4)**			**Success (Week 6)**				**Success (overall)**			
**Group**	**Yes**	**No**	**Total**	**Group**	**Yes**	**No**	**Total**	**Group**	**Yes**	**No**	**Total**
Sham	4	16	20	Sham	6	14	20	Sham	7	13	20
Active	12	8	20	Active	11	9	20	Active	13	7	20
Total	16	24	40	Total	17	23	40	Total	20	20	40

To evaluate the direction and strength of the relationship between questionnaire results and video coding results, variables were aligned as shown in Table 6 and correlations were calculated for each pair of variables at Baseline, weeks 4 and 6 (Table 7). Correlations were somewhat inconsistent between Baseline, week 4 and 6, but were generally weakest at week 4. The best alignment for between questionnaire results and video coding data was for destructive behavior and overall improvement at week 6. The data were sparse for urination and defecation. “Vocalization” showed a moderately positive correlation with “Barks per Minute” and with “Whining.”

**Table 6:** Variables used to compare the video data and questionnaire data.

**Video Data**	**Questionnaire Data**
Destructive/active (DE)	Destructive
Rearranging behavior (RA)	Rearranging
Barks per minute	Vocalization
Whining/howling (WH)	Vocalization
Elimination of urine	Urination
Elimination of feces	Defecation
All Positive (Interacting with the environment + Passive behavior)	Overall
All Negative (Destructive/active + Rearranging behavior + Restlessness/pacing + Orienting to the environment)	Overall

**Table 7:** Correlations between the video data and questionnaire data, separated by time (Baseline, week 4, and week 6).

		**Pearson's correlation coefficient**		
**Video Data**	**Questionnaire Data**	**Week 0**	**Week 4**	**Week 6**
DE	Destructive	0.503 (*n* = 38, *p* = 0.001)	0.159 (*n* = 40, *p* = 0.326)	0.446 (*n* = 39, *p* = 0.004)
RA	Rearranging	0.345 (*n* = 38, *p* = 0.034)	0.248 (*n* = 40, *p* = 0.122)	0.068 (*n* = 39, *p* = 0.681)
Barks per Minute	Vocalization	0.302 (*n* = 38, *p* = 0.065)	0.336 (*n* = 40, *p* = 0.034)	0.202 (*n* = 39, *p* = 0.217)
WH	Vocalization	0.367 (*n* = 38, *p* = 0.024)	0.267 (*n* = 39, *p* = 0.100)	0.376 (*n* = 38, *p* = 0.020)
Urine	Urination	−0.039 (*n* = 38, *p* = 0.816)	0.094 (*n* = 40, *p* = 0.564)	−0.063 (*n* = 39, *p* = 0.705)
Feces	Defecation	0.460 (*n* = 38, *p* = 0.004)		−0.056 (*n* = 39, *p* = 0.736)
All Positive (IE + PA)	Overall	−0.058 (*n* = 38, *p* = 0.729)	−0.174 (*n* = 40, *p* = 0.283)	−0.375 (*n* = 39, *p* = 0.019)
All Negative (DE + RA + RP + OE)	Overall	0.058 (*n* = 38, *p* = 0.727)	0.170 (*n* = 40, *p* = 0.293)	0.375 (*n* = 39, *p* = 0.019)

*Pearson's (linear) correlation, the number of dogs with both data points available, and a p-value comparing the correlation to zero are shown for correlations at each timepoint*.

Exploratory analysis on treatment success was performed after unblinding, Results of the repeated-measures model for dogs in the active treatment group found no significant effects of age, sex, or weight for the percent of time spent in positive behavior, negative behavior, or success on questionnaire results.

### Adverse Events

Treatment was generally well tolerated by dogs. Over the course of the study, 8 adverse events were reported in 8 dogs; all resolved with or without intervention and most were unlikely to be related to the treatment. Two dogs (both on active treatment) vomited once each during the six weeks, one dog had diarrhea that was treated with propectalin and metronidazole (active), one dog had ocular discharge that was treated with ofloxacin (active), one dog fell down stairs and broke a tooth (sham) while a second broke his tooth on his crate (sham). One dog on active treatment came home from daycare with a dry cough and was treated for Bordetella infection. One dog on active treatment jumped off the couch near the end of his study and was described as having a stiffened posture and abnormal gait; this lasted <1 h, resolved without intervention, and did not recur. No other adverse events were reported by owners.

## Discussion

The results of this study support the hypothesis that pulsed electro-magnetic field (PEMF) treatment with this device is effective for the treatment of SA in dogs. The treatment was well tolerated by dogs, and offers an exciting additional tool for veterinarians and owners with dogs suffering from this condition. While questionnaire results were not different between active and sham-device groups, video results showed a significantly larger decrease in negative behaviors in the active device group by 6 weeks, and a higher number of successes, defined as an increase of 100% or greater in positive behaviors, in the active device group by week 4 and overall.

There are several possible reasons for the discrepancy between the questionnaire results and the video results. First, many of the dogs in this study spent a substantial percentage of their time while home alone in the behavioral state of “oriented to the environment.” This is similar to other studies of dogs with SA (6–8). As owners must necessarily be away from their dogs during these separations, it is difficult to appreciate change in this domain without having access to videos. Previous studies have demonstrated a moderate correlation between owner questionnaires and behaviors captured on video when dogs are home alone, with the highest correlations for more easily measurable behaviors (8, 30). In a study by van Rooy et al. (30) the correlation between a standardized questionnaire (Canine Behavioral Assessment and Research Questionnaire; CBARQ) and videos of dogs left home alone found the highest correlations for destructiveness (*r* = 0.70), similar to our findings here (30). In that study, restlessness, agitation, and pacing had the lowest correlation with CBARQ results (*r* = 0.24); hypervigilance/orienting to the environment was the most frequent anxiety behavior noted on video, but had no corresponding CBARQ question for comparison (30). Further, several comments by owners in our study indicated that our questionnaires may not have been sensitive enough or capturing the right measure to reflect the changes they observed. For example, comments from owners of dogs in the active group included two owners who noted that overall demeanor had improved and that their dogs had become less anxious outside the crate and in preparation to go into the crate, and one who commented on decreased whining and waiting during short departures.

The second possible reason for the discrepancy is a caregiver placebo effect, as the improvement in overall scores was seen in both groups. Caregiver placebo effects (where a caregiver reports a change in an outcome measure while their pet is on placebo) are seen in many studies with behavioral outcomes and proxy assessments (31, 32). Specific to studies of treatment for SA, placebo effects were seen in both clinical trials for the approved psychotherapeutics. In the trial for fluoxetine, the incidence of improvement (defined as a one-point change in global score) was seen for 43% of patients receiving a placebo at week 4, and 51% of patients receiving a placebo at week 6 (11). Similar results were found in the study of clomipramine, with improvement in owner global scores in 29, 57, and 62% of patients taking placebo at weeks 4, 8, and 14, respectively (10). The limitations inherent in owner questionnaires of behaviors they are unable to witness are clear. While time-intensive, the video data provide a better representation of the change, or lack of change, in the behaviors of the dogs when left home alone. In the current study, the video data were coded and analyzed with all study investigators and statistician remaining blind, providing further confidence in the results. Future studies could also involve the use of biomarkers, such as salivary vasopressin, to evaluate efficacy, provided additional work has been done to support their use (33).

Other studies of treatments for SA have included a behavior modification plan either as a stand-alone (3, 34, 35) or in conjunction with a behavioral medication (10, 11, 36). As a sole treatment in a small number of dogs, systematic desensitization to separation from their owner was found to improve signs of SA, however speed of improvement was variable across the number of trial separations (35) and previous work has suggested that while it might be effective, there is often low owner compliance for desensitization treatment for SA (13). No specific plan was defined in the fluoxetine trial (11); however, a simple behavioral treatment plan was provided in the clomipramine trial and was evaluated for effect in the main trial (10) and follow-up (36). The authors of the clomipramine studies concluded that the behavioral therapy alone was effective, and that treatment with clomipramine decreased the time to improvement and increased the chance of further improvement even after stopping clomipramine; they posited that clomipramine worked synergistically with the behavioral plan to improve signs of SA. In this study, we included a basic program of “Be positive,” “Reward calm,” “Avoid drama,” and “Reward independence,” but no tailored behavioral modification plan was provided. These suggestions could have contributed to the effect seen in the sham group as discussed above, but also suggests that a behavioral plan, in conjunction with treatment with this PEMF device, could further increase efficacy; however, this needs to be evaluated in future trials. In addition, dogs were eligible for inclusion if they were on one psychoactive medication, provided that the dose was stable for at least 1 month, signs were severe enough to meet entry criteria, and no dose changes were allowed during the study (thus ensuring that any changes seen were not due to changing efficacy of the medication alone). Dogs were not randomized based on medication, and more dogs were receiving medication in the active group than the sham group. Being on psychoactive medication did not affect the distribution of dogs categorized as “successes,” however, synergistic effects cannot be ruled out and should also be evaluated in future trials.

Of note, the high number of dogs whose owners believed they had SA but failed the screening video is important for veterinarians to be aware of. There has been speculation that due to the high percentage of time dogs with SA spend in “orientation to the environment,” the prevalence may be underestimated as owners may not be aware of their dog's behavior when alone. However, it is also possible that many dogs are misdiagnosed with SA if video is not captured as part of the minimum database for diagnosis. As mentioned in previous studies (6–8, 30), our results support the need for capturing video of dogs suspected of having SA prior to making a diagnosis and instituting a treatment plan.

Limitations to our study merit discussion. First, we selected dogs without comorbid noise aversion, storm anxiety, or confinement distress. The overlap between SA and noise aversion is high with studies showing that between 43 and 50% of dogs with SA will show signs of noise aversion (4, 37, 38). Confined dogs with SA displayed stress signs—lip licking—more frequently than unconfined dogs with SA (6). While there are more dogs with SA who do not have storm or noise aversion than vice versa (37, 38), this criterion excluded many dogs from the study who would otherwise have qualified. This decision was made to be able to specifically evaluate efficacy in SA, however it limits our generalizability without further evaluation in this population. In addition, our recruitment period spanned the period when many areas in the United States instituted restrictions due to COVID-19. While this affected only the last few months of recruitment and testing, these restrictions meant changes in owner schedules and the frequency with which some dogs were being left home alone, thus extending the time for recruitment of dogs and owners who met the inclusion criteria and maintained regular schedules for departures despite new restrictions. Next, while uncommon, some missing video could not be analyzed, and inter-rater agreement for one of our variables was poor. The videos that were short also tended to be the ones where the dogs were not visible. One reason for this was an unannounced software update for the cameras that caused the recording to shut off if motion was not detected. As soon as this was discovered, cameras were switched or reset, however some video was lost. Still, the mean duration of video remained high and this affected very few videos. Poor reliability was found for the variable “interacting with the environment;” this is due in part to the small amount of time dogs spent in this behavioral state, in agreement with previous work (8). This was mitigated by having one individual code all three videos for each dog for analysis. Finally, as types of PEMF devices vary in their frequency and output, this study can be generalized only to this type of PEMF device, not to PEMF devices more broadly.

In conclusion, this study found evidence supporting efficacy of this PEMF device in the treatment of canine SA. The treatment was well-tolerated by dogs, and was safe even when combined with a psychotherapeutic. While no individually-tailored behavior modification plan was included in this study, this would likely further improve management of signs. There is some concern that the prevalence of SA will increase following the COVID-19 pandemic, as owners begin to go back to work and dogs who have been adopted during this time will not have had experience being left alone. For many dogs, even those with prior experience being left alone, disruptions to routines and changes in the home may be unsettling, increasing the need for veterinarians to be screening for SA and for development of additional treatment options. This study establishes this PEMF device as an additional tool in our toolbox for management of SA in dogs.

## Data Availability Statement

The data generated from this study are available from Assisi Animal Health but restrictions apply to the availability of these data, which were used under license for the current study, and so are not publicly available. Data are however available from the authors upon reasonable request and with permission of Assisi Animal Health. Requests to access the datasets should be directed to megruen@ncsu.edu.

## Ethics Statement

The animal study was reviewed and approved by NCSU Institutional Care and Use Committee (Protocol #18-174-O). Written informed consent was obtained from the owners for the participation of their animals in this study.

## Author Contributions

KP, JK, and MG designed the study. KP, MG, CE, and BJ conducted the study, collected data, and provided quality control over the data. MG and EG analyzed the data. KP and MG drafted the manuscript. KP, JK, CE, BJ, EG, and MG provided input on the manuscript. All authors contributed to the article and approved the submitted version.

## Funding

This study received funding from Assisi Animal Health and a grant from NC State University awarded to KP and MG (Comparative Medicine Institute New Investigator Clinical Research Award).

## Conflict of Interest

JK is employed by Assisi Animal Health. The study received funding, in part, by Assisi Animal Health. The funder had the following involvement with the study: JK participated in the design of the study, review of data, and review of manuscript. The remaining authors declare that the research was conducted in the absence of any commercial or financial relationships that could be construed as a potential conflict of interest.

## Publisher's Note

All claims expressed in this article are solely those of the authors and do not necessarily represent those of their affiliated organizations, or those of the publisher, the editors and the reviewers. Any product that may be evaluated in this article, or claim that may be made by its manufacturer, is not guaranteed or endorsed by the publisher.

## Footnotes

^1^Calmer Canine, Assisi Animal Health; U.S. Patent No. 11,071,876 B2.
