# Supplementary material for: Randomized, Placebo-Controlled Prospective Clinical Trial Evaluating the Efficacy of the Assisi Anti-anxiety Device (Calmer Canine) for the Treatment of Canine Separation Anxiety
Source: Front Vet Sci. 2021 Dec 20;8:775092. doi: 10.3389/fvets.2021.775092 (PMC8720769; doi:10.3389/fvets.2021.775092)
Supplement: Supplementary file 1 [file Data_Sheet_1.docx]

Supplementary Material

**Supplementary Table 1**. Survey results for distribution of successes across treatment groups (sham/active) and two-sided p-value (Fisher’s exact test). Success for individual behaviors was defined as a two-point improvement in score (each behavior was scored on a 0-4 scale) while a sum score was used for owner-specified behaviors (maximum 12 points) so success was defined as an improvement of at least four points.

|  | Success (sham/active) | Total (sham/active) | p-value |
| --- | --- | --- | --- |
| Destruction | 1/1 | 20/20 | 1.00 |
| Rearranging | 1/1 | 20/20 | 1.00 |
| Vocalization | 4/5 | 20/20 | 1.00 |
| Urination | 1/0 | 20/20 | 1.00 |
| Defecation | 1/1 | 20/20 | 1.00 |
| Overall | 3/0 | 20/20 | 0.231 |
| Owner specified behaviors | 9/11 | 18/19 | 0.746 |

**Supplementary Table 2**. Reliability of behavioral coding. Intraclass coefficients for each behavioral state and event and shown. These are interpreted as poor (<0.5), moderate ( 0.5-0.75), good (0.75-0.90) and excellent (>0.90).

| **Behavioral state/event** | **Intraclass coefficient** | **Interpretation** |
| --- | --- | --- |
| Destructive | 0.92 | Excellent |
| Rearranging | 0.85 | Good |
| Restless/pacing | 0.67 | Moderate |
| Interacting with the environment | 0.45 | Poor |
| Passive | 0.90 | Excellent |
| Oriented to the environment | 0.92 | Excellent |
| Not visible | 0.81 | Good |
| Whining | 0.97 | Excellent |
| Bark | 0.97 | Excellent |
| Yawn | 0.56 | Moderate |
