# Supplementary material for: Randomized, Placebo-Controlled Prospective Clinical Trial Evaluating the Efficacy of the Assisi Anti-anxiety Device (Calmer Canine) for the Treatment of Canine Separation Anxiety
Source: Front Vet Sci. 2021 Dec 20;8:775092. doi: 10.3389/fvets.2021.775092 (PMC8720769; doi:10.3389/fvets.2021.775092)
Supplement: Supplementary file 2 [file Data_Sheet_2.docx]

Supplementary Figure 1. A schematic of the PEMF device (A) and positioning during treatment (B). Active and sham devices were visually identical.


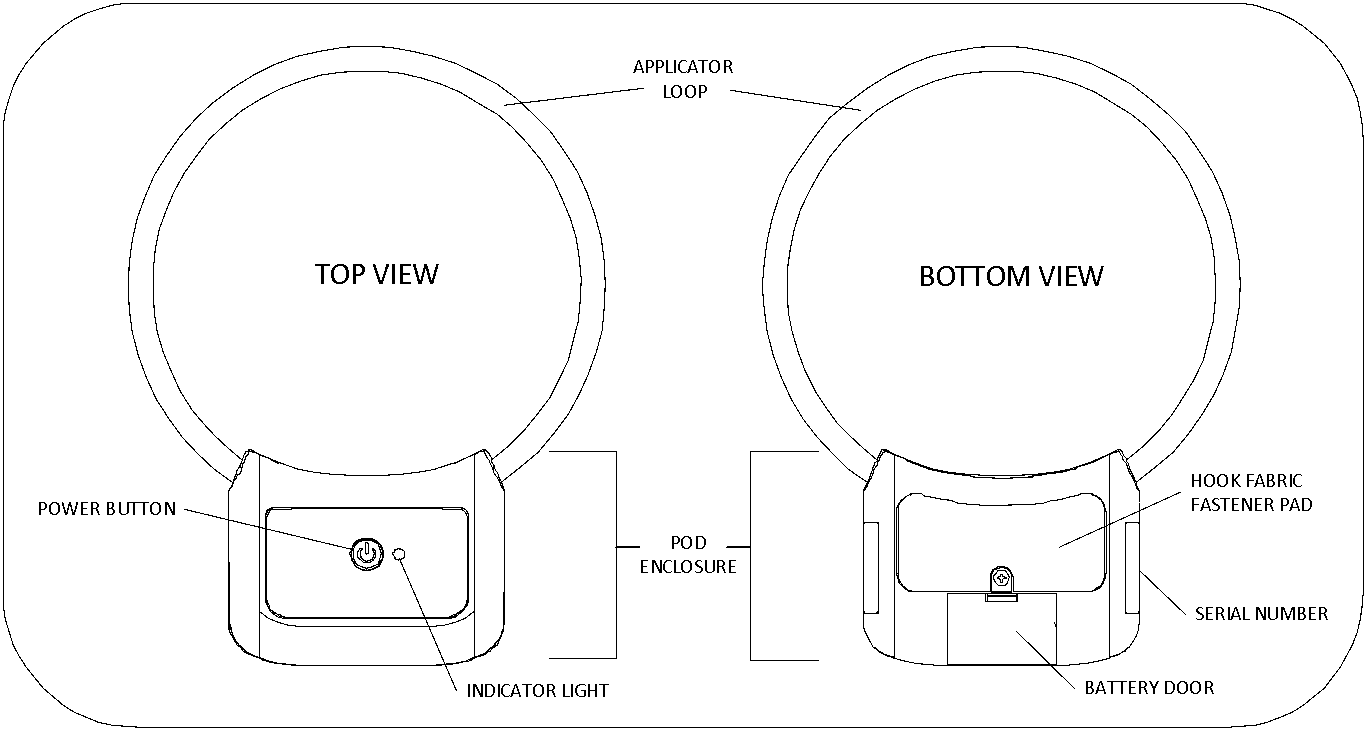


**A**.


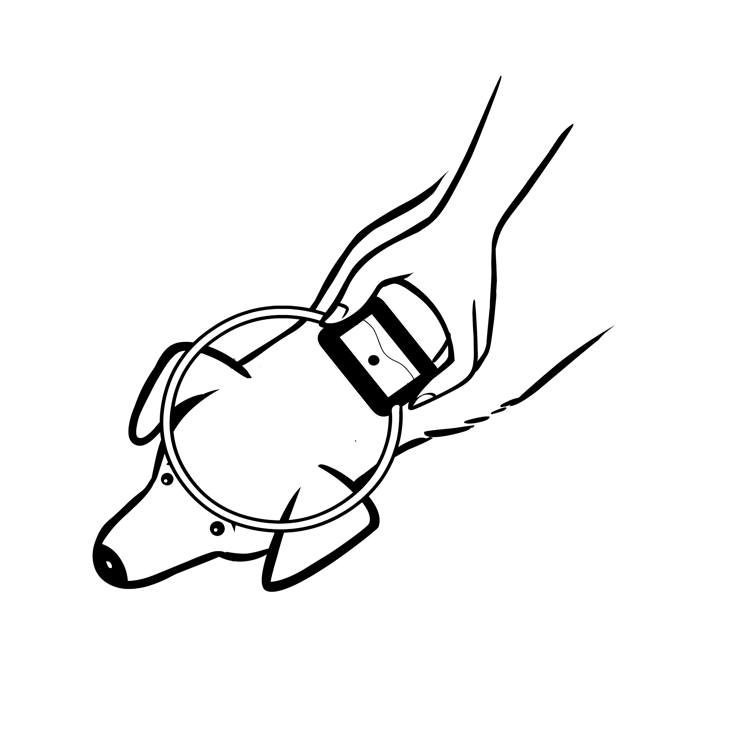


**B.**
